# Personal

**Published:** 1914-05

**Authors:** 


### PERSONAL.

---

Announcement of removal, travel, and other matters of interest are requested. Please report errors in the listing of any physician in the State and other directories, that we may co-operate with the State Society in securing a correct list.

---

Dr. M. Axford of Buffalo has moved to 351 Lafayette ave nue.

---

The office of Dr. Irving R. Johnson of Buffalo was robbed April 1.

---

Dr. Burt C. Johnson of Buffalo returned from a trip to Nassau in April.

---

Dr. Z. J. Lusk of Warsaw was in Cleveland and Pittsburg late in April.

---

Dr. F. W. Scott of Medina has recovered from a severe attack of amydalitis.

---

Dr. F. S. Burlingham of Friendship has been elected Chief of the Fire Department.

---

Dr. James A. Gibson of Buffalo has moved to the Melton Manor on Chapin Parkway.

---

Dr. E. A. Sharp of Buffalo announces the removal of his office to 481 Franklin Street.

---

Dr. Herman E. Hayd of Buffalo returned about the middle of April from a ten months' trip to the Orient.

---

Dr. George P. Michel of Buffalo had his automobile damaged by a collision with a street car April 9, escaping himself with slight injuries.

---

Dr. E. C. Register, for 25 years editor of the Charlotte Medical Journal, has been elected president of the Tri-State Medical Society of Virginia and the Carolinas.

---

Dr. S. J. Brown of Mt. Morris has been elected Health Officer of the town, vice Dr. G. C. Fiske, who has moved to Buffalo. He also succeeds the latter as surgeon to the Lackawanna R. R.

Dr. F. C. Goldsborough of Buffalo has moved to the residence which he recently purchased, 515 Franklin Street.

Dr. Regina Flood Keyes of Buffalo announces the removal of her residence to the Buckingham Hotel, and of her office to the Professional Building, Allen Street and Irving Place.

The following physicians attended the annual banquet of the University of Rochester Alumni, Buffalo Association, April 4: F. Parke Lewis, Irving M. Snow, Wm. H. Thornton, H. K. DeGroat, Lesser Kaufmann, E. A. Rhodes, A. L. Benedict, of Buffalo; John W. Le Seuer of Batavia.

